# 3D reconstructed brain images reveal the possibility of the ogg1 gene to suppress the irradiation-induced apoptosis in embryonic brain in medaka (*Oryzias latipes*)

**DOI:** 10.1093/jrr/rrac005

**Published:** 2022-03-12

**Authors:** Takako Yasuda, Duolin Li, Erge Sha, Fumitaka Kakimoto, Hiroshi Mitani, Hiroshi Yamamoto, Tomoko Ishikawa-Fujiwara, Takeshi Todo, Shoji Oda

**Affiliations:** Laboratory of Genome Stability, Department of Integrated Biosciences, Graduate School of Frontier Sciences, The University of Tokyo, Kashiwa, Chiba 277-8562, Japan; Center for Environmental Risk Research, National Institute for Environmental Studies, Onogawa 16-2, Tsukuba, Ibaraki 305-8506, Japan; Laboratory of Genome Stability, Department of Integrated Biosciences, Graduate School of Frontier Sciences, The University of Tokyo, Kashiwa, Chiba 277-8562, Japan; Laboratory of Genome Stability, Department of Integrated Biosciences, Graduate School of Frontier Sciences, The University of Tokyo, Kashiwa, Chiba 277-8562, Japan; Laboratory of Genome Stability, Department of Integrated Biosciences, Graduate School of Frontier Sciences, The University of Tokyo, Kashiwa, Chiba 277-8562, Japan; Laboratory of Genome Stability, Department of Integrated Biosciences, Graduate School of Frontier Sciences, The University of Tokyo, Kashiwa, Chiba 277-8562, Japan; Center for Environmental Risk Research, National Institute for Environmental Studies, Onogawa 16-2, Tsukuba, Ibaraki 305-8506, Japan; Department of Radiation Biology and Medical Genetics, Graduate School of Medicine, Osaka University, B4, 2-2, Yamadaoka, Suita, Osaka 565-0871, Japan; Department of Radiation Biology and Medical Genetics, Graduate School of Medicine, Osaka University, B4, 2-2, Yamadaoka, Suita, Osaka 565-0871, Japan; Laboratory of Genome Stability, Department of Integrated Biosciences, Graduate School of Frontier Sciences, The University of Tokyo, Kashiwa, Chiba 277-8562, Japan

**Keywords:** ogg1, apoptosis, medaka, irradiation, 3D reconstruction, embryonic brain

## Abstract

The accumulation of oxidative DNA lesions in neurons is associated with neurodegenerative disorders and diseases. Ogg1 (8-oxoG DNA glycosylase-1) is a primary repair enzyme to excise 7,8-dihydro-8-oxoguanine (8-oxoG), the most frequent mutagenic base lesion produced by oxidative DNA damage. We have developed ogg1-deficient medaka by screening with a high resolution melting (HRM) assay in Targeting-Induced Local Lesions In Genomes (TILLING) library. In this study, we identified that ogg1-deficient embryos have smaller brains than wild-type during the period of embryogenesis and larvae under normal conditions. To reveal the function of ogg1 when brain injury occurs during embryogenesis, we examined the induction of apoptosis in brains after exposure to gamma-rays with 10 Gy (137Cs, 7.3 Gy/min.) at 24 h post-irradiation both in wild-type and ogg1-deficient embryos. By acridine orange (AO) assay, clustered apoptosis in irradiated ogg1-deficient embryonic brains were distributed in a similar manner to those of irradiated wild-type embryos. To evaluate possible differences of gamma-ray induced apoptosis in both types of embryonic brains, we constructed 3D images of the whole brain based on serial histological sections. This analysis identified that the clustered apoptotic volume was about 3 times higher in brain of irradiated ogg1-deficient embryos (*n* = 3) compared to wild-type embryos (n = 3) (*P* = 0.04), suggesting that irradiation-induced apoptosis in medaka embryonic brain can be suppressed in the presence of functional ogg1. Collectively, reconstruction of 3D images can be a powerful approach to reveal slight differences in apoptosis induction post-irradiation.

## INTRODUCTION

DNA and cells of the human body are constantly exposed to oxidative damage from both exogenous and endogenous sources. The accumulation of oxidative damage may lead to cellular transformation and ultimately to the development of tumors, a variety of other pathophysiological states and aging syndromes [1, 2]. Non-bulky oxidative DNA lesions are mainly repaired via base excision repair (BER) pathway, which is initiated by DNA glycosylases such as 8-oxoguanine DNA glycosylase (ogg1) [3, 4]. The most frequent base lesion, 7,8-dihydro-8-oxoguanine (8-oxoG) is one of the most commonly formed oxidative DNA lesions in the cell. Since it mis-pairs with A during replication, 8-oxoG is also a highly mutagenic lesion, producing G:C to T:A transversions. Ogg1 is a primary repair enzyme responsible for identification and excision of 8-oxoG lesions [5, 6].

The brain is one of the organs especially vulnerable to oxidative damage because of its high oxygen demand and abundance of peroxidation-susceptible lipid cells [7, 8]. Mutations in the ogg1 gene have been implicated as a key factor in acceleration of neurodegenerative diseases such as Alzheimer’s disease [9, 10] and Parkinson’s disease [11, 12]. Above all, the developing brain is particularly vulnerable to oxidative damage, which may produce a variety of DNA lesions and lead to brain impairment [13, 14]. While it is generally postulated that the importance of the ogg1 gene in maintaining the physiological function of the developing brain, how ogg1 dysfunction affects susceptibility to brain impairment during neurogenesis is yet largely unknown. It has been known that exposure to ionizing radiation (IR) is one of the exogenous sources of oxidative damage to biomolecules such as DNA, proteins, and lipids since it leads to the generation of reactive chemical species that alter the atomic structure through direct interactions of radiation with target macromolecules or via products of water radiolysis [15, 16]. We have therefore investigated the role of ogg1 after exposure to IR using embryonic medaka brain as a vertebrate model.

To date, medaka is a useful model organism as with zebrafish which is the most widely used laboratory fish, for investigating genome stability and gene function due to their ease of handling and large numbers of progeny per generation. In particular, embryos develop outside the mother’s body, enabling easy visual observation for their translucent embryos [17, 18]. We previously investigated the detrimental effects of gamma-ray irradiation on the developing brain and found that the embryonic medaka at 3 days post-fertilization (3 dpf) which is equivalent to the developmental stage 28 [19] exhibited the highest frequency of apoptosis induction post-irradiation throughout its life, especially in the neural proliferating area of the optic tectum (OT) due to its high sensitivity [20–22]. In this study, we have developed ogg1-deficient medaka by screening with the high resolution melting (HRM) assay which was established by Ishikawa et al. [23] in Targeting-Induced Local Lesions In Genomes (TILLING) library. We report here a novel role for ogg1 in the maintenance of developing medaka brain focusing on the 3 dpf (developmental stage 28) at 24 h after irradiation when the most frequent apoptotic induction was occurred [24, 25] by evaluating possible differences of IR-induced apoptotic frequency between wild-type and ogg1-deficient embryonic brains based on 3D reconstructed images.

## MATERIALS AND METHODS

### Screening of ogg1 mutants in TILLING library

A gene-driven mutagenesis library has sets of frozen sperm and genomic DNA that were prepared from 5771 F1 males. Each genomic DNAs within 5771 in the library was amplified by PCR with ogg1 specific primers, 3 different amplicons covering exon 2 and 3 (F: ATGTTGCGTCTCATCCCG, R: CAGCTCTGTGAAACCTGTACT; F: CCTGAATACTGCTTTGCCTCTA, R: TCTGGAGATGCAATGCACGAA; F: GCCGTTGAGTCAGAGTGATG, R: TGACACAAACGGGACAAAAA). PCR reactions were performed in 96-well microtiter plates in 7-μL volumes. Reactions included 7 ng of genomic DNA in 1 × KOD Plus PCR buffer (Toyobo, Tokyo), with 1 mM MgSO4, 200 μM of deoxynucleotide triphosphate, 0.6 × LC Green PLUS (Idaho Technology), 0.14 U of KOD Plus polymerase (Toyobo, Tokyo), and 300 nM of each primer. Reactions were overlaid with 10 μL of mineral oil (Nakalai, Kyoto, Japan), the plates were centrifuged (1500 × g for 3 min), and PCR was performed in an iCycler (Bio-Rad Laboratories, Hercules, CA, USA) or PXE0.2 Thermal Cycler (Thermo Scientific, Rockford, IL, USA). The cycling and melting conditions were set as follows: one cycle of 94°C for 2 minutes, followed by 45 cycles of 94°C for 15 seconds; a primer-specific annealing temperature for 25 seconds, and then 68°C for 25 seconds; and a final denaturing and re-annealing step (one cycle of 94°C for 30 seconds followed by rapid cooling to 28°C). The final step was included to redistribute DNA strands derived from mutant and wild-type alleles to maximize heteroduplex formation. After PCR, the plates were centrifuged (1500 × g for 3 min) and imaged in a 96-well Light Scanner (Idaho Technology Inc., Salt Lake City, UT, USA) for HRM analyses as previously described [23, 26].

The genomic DNAs showing melting curves that differed from those of the wild-type allele were selected as mutant candidates. The selected genomic DNAs were purified using a HRM amplicons with a Sephadex G50 (Fine DNA Grade) column and then used as a template for the sequencing reaction. Sequencing reactions were carried out by using BigDye Terminator version 3.1 (Applied Biosystems, Foster City, CA) and the ABI 3730xl sequencing platform.

### Fish and embryos

Hd-rR inbred strain medaka (*Oryzias latipes*) [27] and ogg1-deficient medaka which was established in this study were used. The fish were kept at 26–28°C under a 14-hr light and 10-hr dark cycle and were fed brine shrimp (*Artemia franciscana*) and powdered diet (TetraMin, Tetra Werke, Melle, Germany) three times per day. Egg clusters were collected and rubbed between two small pieces of paper towel to remove filaments on the chorion; the isolated eggs were then incubated in a Petri dish filled with 7 ml of tap water containing 0.00001% (w/v) methylene blue at 26–28°C. The collected eggs at 72 hr after incubation were examined the development according to Iwamatsu [19] under stereomicroscope (Leica M125, Nussloch, Germany). We selected the embryos at stage 28 (3 dpf).

### Irradiation

Embryos at stage 28 (3 dpf) were irradiated with 10 Gy gamma-rays in The University of Tokyo emitted from 137Cs (Gammacell 3000Elan, MDS Nordion, Ottawa, Canada) at a dose rate of 7.3 Gy/min at room temperature in a plastic tub with water. The embryos were exposed to gamma rays with only a limited dose, 10 Gy.

### Identification of IR-induced apoptosis by AO-assay

Acridine orange (AO) (Sigma-Aldrich, St. Louis, MO, USA), a single-strand DNA intercalating vital dye, selectively stains the nuclei of apoptotic cells without labeling necrotic cells [28, 29]. To confirm IR-induced apoptosis in the whole brain at 24 h after the irradiation, the irradiated embryos were stained with AO (17 μg/ml) as previously described [24]. The AO-stained apoptosis in the whole brain were identified and images were captured with consecutive optical sections at 10 μm intervals from the surface to the bottom with total 15 slices by a confocal laser scanning microscope (FV3000, Olympus, Tokyo, Japan).

### Histopathological analysis

For histopathological analyses in embryonic brain, we prepared three irradiated samples and sham controls in wild-type and ogg1-deficient embryos. Embryos were fixed in 4% (w/v) paraformaldehyde in 0.1 M phosphate buffer overnight at 0–4°C. The fixed samples were dehydrated in a graded ethanol series, then embedded in plastic resin (Technovit 8100, Heraeus Kulzer, Wehrheim, Germany). Cross-sections were cut at 8 μm thickness and stained with cresyl violet as previously described [24]. The sections were examined under light microscopy (BX50, Olympus, Tokyo, Japan) and captured images with digital camera (DFC7000T, Leica Microsystems, Wetzlar, Germany).

### Measurement of the maximum width of the optic tectum

To evaluate the brain size quantitatively under normal conditions, we measured the maximum width of the optic tectum (OT), which is the largest subdivision of medaka brain at 4 dpf, 7 dpf and 12 dpf (*n* = 6, respectively). Our previous findings indicated that the measured value of maximum width of the OT could be used as a useful tool for evaluation of brain size [20]. The fixed embryos with 4% paraformaldehyde in 0.1 M phosphate buffer for 1 day were mounted on a glass slide for measurement under a microscope (BX50, Olympus, Tokyo, Japan). Images were captured with a digital camera (DFC7000T, Leica, Wetzlar, Germany).

### Reconstruction and computer visualization of 3D images of the whole brain

Serial histological sections were analyzed with a microscope (BX50, Olympus, Tokyo, Japan) and captured with a digital camera (DP70, Olympus, Tokyo, Japan). Serial images were imported into Reconstruct software [30] for 3D reconstruction. After adjusting pixel size and alignment, the whole brain as well as clustered apoptotic neurons on each section were manually traced and automatically reconstructed into three-dimensional images. The above instructions are summarized as schematic images in Supplementary Fig. 1.

### R analysis data

The size and number of clustered apoptotic cells in the whole brain between wild-type and ogg1-deficient embryonic irradiated brains were compared and shown in histograms. Moreover, the size distribution of clustered apoptotic cells in the whole brain was calculated by kernel density estimation (KDE) method. Statistical analysis was performed and figures were generated using R statistical software (URL https://www.R-project.org/).

### Statistical analysis

The maximum width of the OT in ogg1-deficient embryos under normal conditions were compared with that in wild-type embryos by the two-tailed student’s t test. In addition, the brain volume of irradiated embryos was compared with that of sham-controls in wild-type and ogg1-deficient embryos by the two-tailed student’s t test. A p^*^ value <0.05 was considered statistically significant and p^*^^*^ < 0.01 was considered highly statistically significant. We summarize the *P*-values of all experiments in Supplementary Table 1.

## RESULTS

**Screening for ENU-induced mutations in the ogg1 gene in a** **medaka TILLING library**

**Fig. 1.**
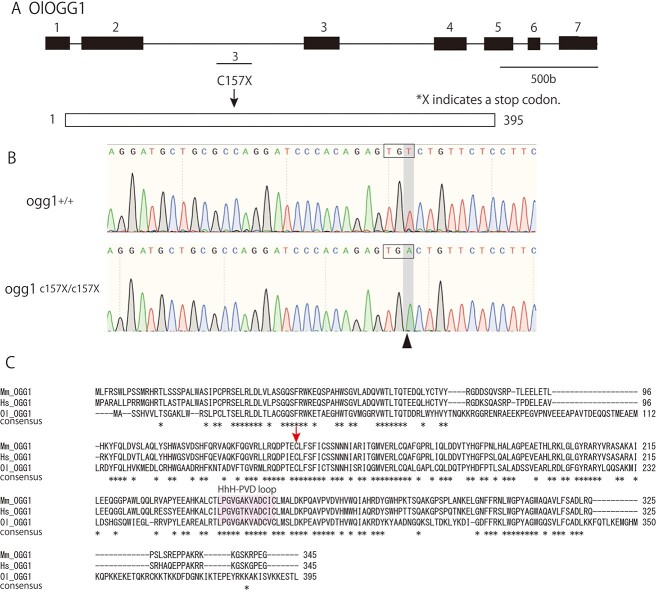
N-ethyl-N-nitrosourea (ENU)-induced mutation identified by Targeting Induced Local Lesions IN Genomes (TILLING) in the *Oryzias latipes* ogg1 gene The medaka ogg1 gene was identified as a single gene orthologue on chromosome 7 in medaka genome. Genome organization and protein coding region in ogg1 are indicated by squared areas in the top; the protein in exon 3 is shown below. Numbers in the top panel refer to exons; numbers in the bottom panel refer to amino acids. The ENU-induced mutation is shown by arrow, which created premature stop codons in the protein (C157X for ogg1). (B) Sequence traces of the region of the point mutation (arrowhead) in the founder fish. (C) Sequence alignment of vertebrate (*Hs, Homo sapiens; Mm, mus musculus; Ol, O. latipes*) in exon 3. Conserved residues are indicated with asterisks below. The arrow indicates the mutation site. The helix–hairpin–helix (HhH)–PVD loop motif, conserved in the DNA endonuclease III (endo III) family of DNA glycosylases, is highlighted in red.

We performed the HRM assay to screen for N-ethyl-N-nitrosourea (ENU) induced mutations in the ogg1 gene with 3 different amplicons covering exon 2 and 3 (for detailed information, see Materials and Methods section). After subsequent sequencing of HRM-positive PCR amplicons, we identified samples 4 and 2 independent mutations with amino-acid-substitution type in exon 2 and 3, respectively. We retrieved highly likely loss-of-function mutations in the ogg1 gene by screening for nonsense mutations and found a point mutation in exon 3 in ogg1 which is present in chromosome 7 (ENSORLG00000010758) and creates premature stop codons in the protein (C157X for ogg1) (arrow in Fig 1A, arrowhead in Fig. 1B). The ogg1 gene contains 395 amino acids (XP_004070580.1) and displays a 51.9% similarity to mouse ogg1 and a 40.3% similarity to human ogg1, with the functional motif (HhH-PVD loop) remarkably similar and conserved among medaka, mice and humans (labeled with red in Fig. 1C). To demonstrate its loss-of-function in nature, heterozygote obtained by artificial insemination in CAB strain was backcrossed five times to wild-type fish, and then homozygous mutant for ogg1 was established by mutual crossing of the heterozygotes.

### The role of medaka ogg1 gene in the developing embryonic brain

**Fig. 2.**
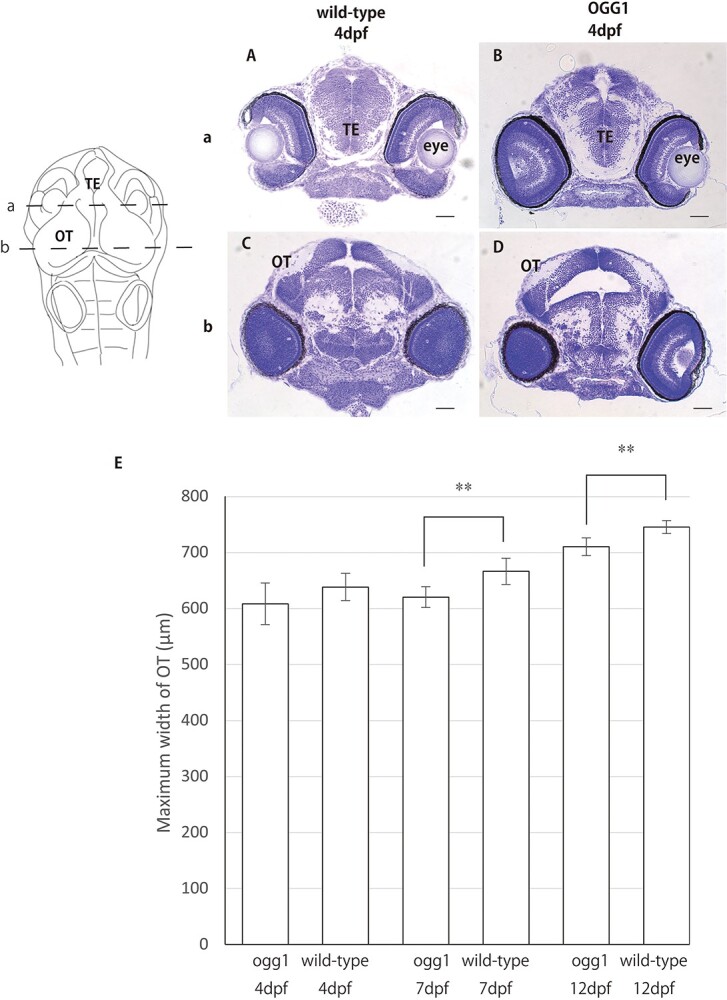
A comparison of the brain size between wild-type and ogg1-deficient medaka during the early period of development Histopathological analysis was performed in the frontal sections with line a and b in a schematic image of medaka embryonic brain as indicated in the upper left side. In ogg1-deficient embryonic brain, morphological abnormalities were not found in the TE (B) and the OT (D) as with wild-type embryonic brain (A and C, respectively) at 4 days post fertilization (dpf). The maximum width of the OT was compared between ogg1-deficient and wild-type embryonic brains at 4 dpf, 7 dpf and 12 dpf. Highly significant differences were found between them at 7 dpf and later in the process of development. A p ^*^ value <0.05 was considered statistically significant and p ^*^^*^ < 0.01 was considered highly statistically significant. OT: optic tectum; TE: telencephalon Scale bar = 50 μm.

Under normal conditions, ogg1-deficient embryos hatch at 6–7 dpf and hatching rate shows 90% (*n* = 38 out of 42), which is almost the same as wild-type embryos (hatching period: 6–7 dpf, hatching rate: 85%; *n* = 33 out of 39). To reveal the role of the ogg1 gene in developing brain in medaka, we performed histopathological analyses in wild-type and ogg1-deficient embryonic brains at 4 dpf. When compared the retinal development in wild-type (Fig. 2A) to ogg1-deficient embryos (Fig. 2B), we found no difference in retinal arrangement between both types of embryos. In addition, in ogg1-deficient embryonic brain, obvious morphological defects were not found in the telencephalon (TE) (Fig. 2B) and the OT (Fig. 2D) comparted to wild type brain (Fig. 2A, C), indicating that no obvious difference was found in brain development between them under non-irradiated conditions. To evaluate brain size quantitatively, we measured the maximum width of the OT in ogg1-deficient and wild-type brains. The maximum width of the OT in ogg1-deficient and wild-type brains were 608 ± 40 μm (mean ± SD) and 638 ± 26 μm (mean ± SD) at 4 dpf, 620 ± 44 μm (mean ± SD) and 666 ± 23 μm (mean ± SD) at 7 dpf, 710 ± 16 μm (mean ± SD) and 745 ± 13 μm (mean ± SD) at 12 dpf, indicating that the brains of ogg1-deficient larvae were significantly smaller than those of wild-type embryos at 7 dpf (*P* = 0.023 by student t test, *n* = 4) and 12 dpf (*P* = 0.021 by student t test, n = 4) (Fig. 2C). These results suggest that the deficiency of the ogg1 gene will cause a developmental retardation in medaka embryonic brain.

**Fig. 3.**
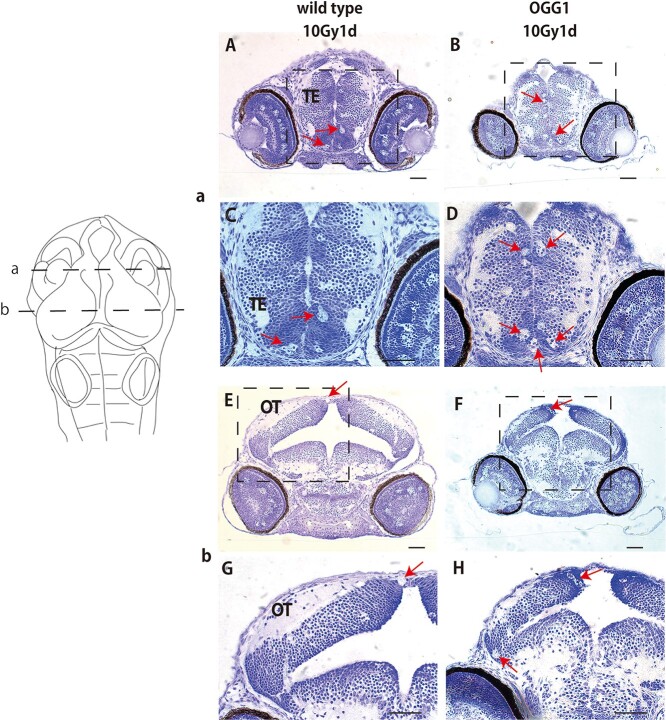
Histopathological analysis to reveal the difference in apoptotic induction between wild-type and ogg1-deficient embryonic brains post-irradiation Histopathological analysis was performed in the frontal sections with line a and b in a schematic image of medaka embryonic brain as indicated in the upper left side. In the TE of ogg1-deficient embryonic brain, a higher number of clustered apoptosis was found (arrows in B and D) as compared to wild-type embryos (arrows in A and C), whereas obvious differences were not identified in the OT between them (arrows in E-H). OT: optic tectum; TE: telencephalon Scale bar = 50 μm.

**Fig. 4.**
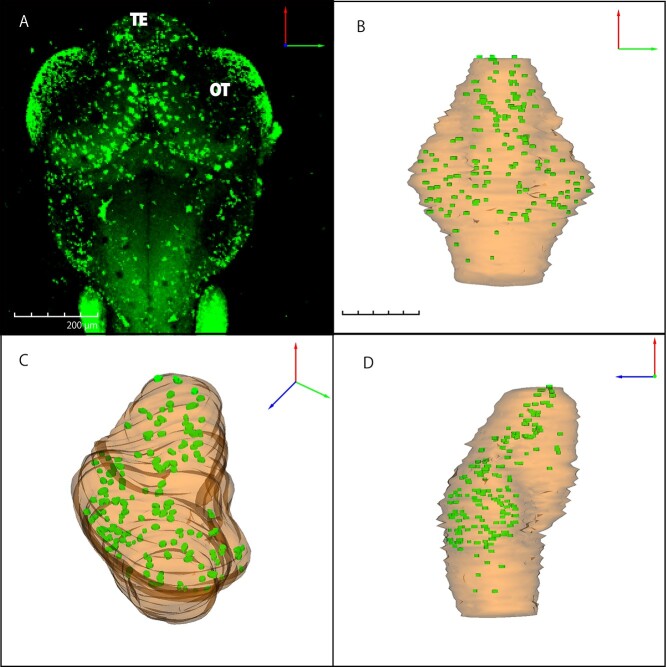
Spatio-temporal distribution of irradiation-induced apoptosis by confocal microscope and 3D reconstructed images in wild-type embryonic brain The whole-brain distribution of AO-stained apoptosis in wild-type embryo at 24 h after irradiation was captured with a confocal laser scanning microscope (A). Reconstructed 3D images for apoptotic distribution were shown from the three different angles; dorsal view (B), slanting view (C) and sagittal view (D). Orange for brain tissue and green for clustered apoptosis. OT: optic tectum; TE: telencephalon Scale bar = 200 μm.

**Fig. 5.**
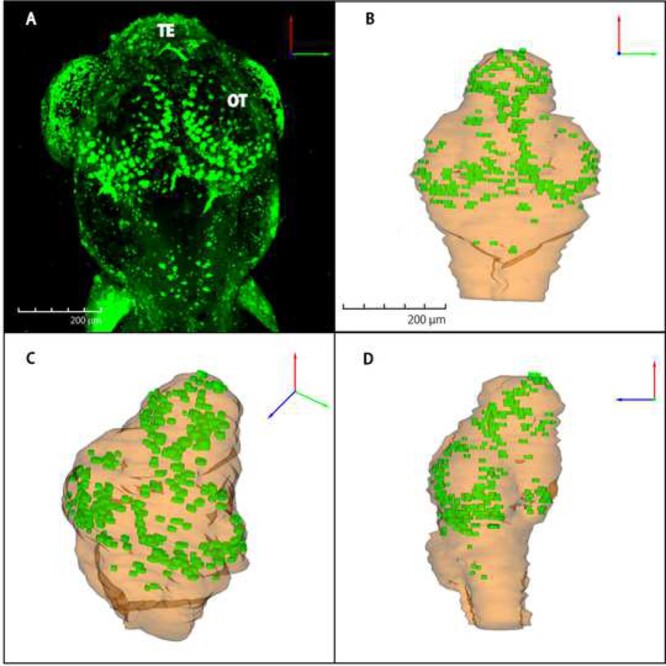
Spatio-temporal distribution of irradiation-induced apoptosis by confocal microscope and 3D reconstructed images in ogg1-deficient embryonic brain The whole-brain distribution of AO-stained apoptosis in ogg1-deficient embryo at 24 h after irradiation was captured with a confocal laser scanning microscope (A). Reconstructed 3D images for apoptotic distribution were shown from the three different angles; dorsal view (B), slanting view (C) and sagittal view (D). Orange for brain tissue and green for clustered apoptosis. OT: optic tectum; TE: telencephalon Scale bar = 200 μm.

**Fig. 6.**
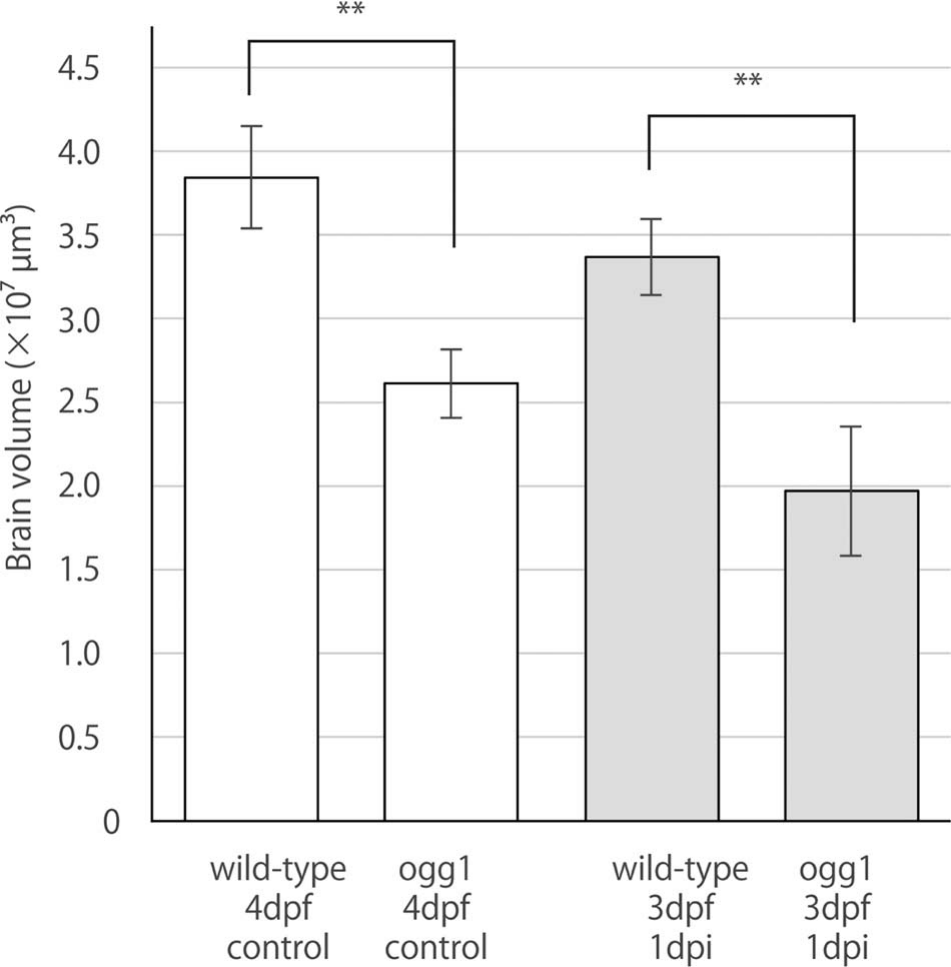
A comparison of brain volume between wild-type and ogg1-deficient embryos under non-irradiated and post-irradiated conditions The analysis of reconstructed 3D images identified that ogg1-deficient embryo have significant smaller brains than wild-type (*P* = 0.004 by student t test) under normal conditions. At 24 h after the irradiation, ogg1-deficient embryonic brain became severely smaller than wild-type with statistically significant difference (*P* = 0.006 by student t test).

***The ogg1 gene suppress the induction of apoptosis after exposure to radiation***

To reveal the function of ogg1 after IR-induced brain injury during embryogenesis, we prepared serial histological sections and examined irradiation-induced apoptosis in ogg1-deficient and wild-type embryos at 24 h after irradiation with gamma-ray (10 Gy). As in our previous reports [24, 31], we found distinct two types of apoptosis by electron microscopic observations, namely single apoptotic cells and clustered apoptotic cells that correspond to rosette-shaped AO-stained apoptosis. In this study, we focused on clustered apoptosis to evaluate the frequency of IR-induced apoptosis quantitatively between wild-type and ogg1-deficient embryos. In the TE, a larger number of clustered apoptosis, were obviously found in ogg1-deficient embryos (arrows in Fig. 3B and D) compared to wild-type embryos (arrows in Fig. 3A and C). Whereas in the OT, significant histopathological differences in apoptotic induction were not identified between them (arrows in Fig. 3E–H). To examine the distribution of irradiation-induced apoptosis in the whole brain, we performed AO-assay at 24 h after the irradiation in both types of irradiated embryos. In confocal images, distributions of AO-positive clustered apoptosis in the brains were similar in irradiated wild-type (Fig. 4A) and ogg1-deficient embryos (Fig. 5A). To further evaluate the differences in spatial distribution of irradiation-induced clustered apoptosis in both type of irradiated embryonic brains, we reconstructed 3D images based on serial histological sections of irradiated and non-irradiated embryonic brains except for eyes (see Materials and Methods) in wild-type (Fig. 4B–D) and ogg1-deficient embryos (Fig. 5B–D). In non-irradiated embryos at 4 dpf, the 3D reconstructed images identified that the brain volume of ogg1-deficient embryo (2.6 10^7^ ± 1.6 × 10^6^ μm^3^, mean ± SD, *n* = 3) was significantly smaller than that of wild-type embryo (3.8 × 10^7^ ± 2.5 × 10^6^ μm^3^, mean ± SD, n = 3) (*P* = 0.004 by student t test, n = 3) (Fig. 6, Table 1). When both types of 3 dpf embryos were irradiated, the brain volume of irradiated wild-type embryo became smaller (3.4 × 10^7^ ± 1.9 × 10^6^ μm^2^, mean ± SD, *n* = 3) than that of non-irradiated wild-type (*P* = 0.10 by student t test, n = 3), whereas ogg1-deficient embryo became smaller intensively (2.0 × 10^7^ ± 3.1 × 10^6^ μm^2^, mean ± SD, n = 3) even though there was no statistical difference from that of non-irradiated ogg1-deficient embryos (*P* = 0.06 by student t test, *n* = 3) (Table 1). The irradiated brain volume of ogg1-deficient embryos became significantly smaller than that of irradiated wild-type embryos (*P* = 0.004 by student t test, *n* = 3) (Fig. 6, Table 1). In non-irradiated embryonic brains, clustered apoptosis was observed only in one of the three embryos. Whereas in ogg1-deficient embryonic brain, we found a few clustered apoptosis among all three embryos in marginal area of OT and TE. The volume of clustered apoptosis in non-irradiated ogg1-deficient embryos (5.6 × 10^3^ ± 2.3 × 10^3^ μm^3^, mean ± SD, *n* = 3) was higher than that in wild-type brain (1.5 × 10^3^ ± 2.6 × 10^3^ μm^3^, mean ± SD, n = 3), even though there was no statistical difference (*P* = 0.06 by student t test) (Table 1). When embryos were exposed to radiation at 3 dpf, 3D reconstructed images

**Table 1:** A comparison of apoptotic volume between wild-type and ogg1-deficient embryonic brains under non-irradiated and 24 h after irradiated conditions

		Volume of Clustered Apoptosis (μm^3^)	Brain Volume (μm^3^)
wild-type_control	sample 1	0	3.93 × 10^7^
	sample 2	4.51 × 10^3^	3.50 × 10^7^
	sample 3	0	4.10 × 10^7^
	mean ± SD	1.50 × 10^3^ ± 2.12 × 10^3^	3.84 × 10^7^ ± 2.52 × 10^6^
ogg1_control	sample 1	4.87 × 10^3^	2.42 × 10^7^
	sample 2	8.07 × 10^3^	2.82 × 10^7^
	sample 3	3.71 × 10^3^	2.60 × 10^7^
	mean ± SD	5.55 × 10^3^ ± 1.84 × 10^3^	2.61 × 10^7^ ± 1.64 × 10^6^
wild-type_IR	sample 1	1.52 × 10^5^	3.28 × 10^7^
	sample 2	2.16 × 10^5^	3.63 × 10^7^
	sample 3	0.43 × 10^5^	3.2 × 10^7^
	mean ± SD	1.37 × 10^5^ ± 0.71 × 10^5^	3.37 × 10^7^ ± 0.19 × 10^7^
ogg1_IR	sample 1	2.93 × 10^5^	1.59 × 10^7^
	sample 2	3.96 × 10^5^	1.96 × 10^7^
	sample 3	5.99 × 10^5^	2.36 × 10^7^
	mean ± SD	4.29 × 10^5^ ± 1.27 × 10^5^	1.97 × 10^7^ ± 3.14 × 10^6^

 identified the spatial distribution of clustered apoptosis in the whole brain at 24 h post-irradiation (Fig. 4B-D) and it occupied 1.4 × 10^5^ ± 0.87 × 10^5^ μm^3^ (mean ± SD, n = 3) in the irradiated wild-type embryonic brain (n = 3) (Table 1). In irradiated ogg1-deficient embryos at 24 h after irradiation, the frequency of clustered apoptosis (Fig. 5B–D) was about 3 times higher than that of irradiated wild-type embryos (Fig. 4B–D), and it occupied 4.3 × 10^5^ ± 1.6 × 10^5^ μm^3^ (mean ± SD, n = 3) with significant differences from irradiated wild-type embryos (*P* = 0.04 by student t test) (Table 1). In addition, to further evaluate the size and the number of irradiation-induced clustered apoptosis in irradiated brains, we performed R software analyses. The histograms indicate that the numbers of clustered apoptosis in all sizes, which are classified from 0 to 560 μm^2^ in increments of 20 μm^2^, for ogg1-deficient embryos were larger (*n* = 894) than those for wild-type embryos (*n* = 335) (Fig. 7A). Considering the ratios of the area in each size class to the total number of clustered apoptosis, especially those in size classes of 280 μm^2^ and larger were all higher in ogg1-deficient embryos than wild-type embryos (which occupied 14% and 3.3% to the total number of clustered apoptosis, respectively) (Fig. 7B).

## DISCUSSION

Since it had been unclear how ogg1 dysfunction affects susceptibility to brain impairment during neurogenesis, we have established ogg1-deficient medaka by screening with the HRM assay in TILLING library and examined the induction of apoptosis in the brain after exposure to gamma-rays with 10 Gy at 24 h post-irradiation both in wild-type and ogg1-deficient embryonic brain. We compared the frequency of IR-induced apoptosis between ogg1-deficient and wild-type embryonic brains by AO-staining, however, obvious differences between them were not found. By histopathological analysis, a larger number of clustered apoptosis were obviously found in ogg1-deficient embryos as compared to wild-type embryos. Consequently, to compare the spatial distribution of IR-induced apoptosis in more detail, we reconstructed 3D images based on serial histological sections. This analysis revealed a significantly higher difference between them in the frequency of IR-induced apoptosis (Table 1). Moreover, R software analyses also indicated that ogg1-deficient embryos induced a higher frequency and a larger area of apoptosis than wild-type embryos (Fig. 7).

Previous research in zebrafish demonstrated that the deficiency of ogg1 caused the morphological defects in the embryonic brain due to excessive induction of apoptosis and decreased proliferation of neuronal cells [32]. Another study in zebrafish reported that no obvious morphological difference was observed between wild-type and ogg1-deficient embryos under normal conditions, however, the ogg1 mutant zebrafish developed more severe lens lesions following oxidative damage [33]. Medaka ogg1-deficient embryos demonstrated no difference regarding hatching period, hatchability and histopathology as compared to wild-type embryos under normal conditions. As with zebrafish embryonic brain, the 3D reconstructed images revealed that ogg1-deficient embryo exhibited a significantly smaller brain size and a higher frequency of spontaneous apoptosis than wild-type under normal conditions (Table 1). Although the difference between the brain volumes of irradiated and non-irradiated ogg1-deficient medaka embryos was not statistically significant (*P* = 0.06), both of histopathological and 3D image reconstruction analyses revealed that gamma-ray irradiation induced a greater number of clustered apoptosis in the brain of ogg1-deficient embryos than wild-type embryos (Fig. 6, Table 1), which might result in the smaller size of the brain of irradiated ogg1-deficient embryos through atrophy of neural tissue and suppression of brain growth. This finding strongly indicated that the analysis utilizing reconstructed 3D images can be a powerful approach to reveal slight differences in induction frequency of apoptosis among gene knock out medaka strains.

Notably, our studies demonstrated that enhanced risks for brain development were exhibited when ogg1-deficient embryo was exposed to one of the exogenous oxidative stressor, namely irradiation. As consistent with our results, neuronal cells in rats exhibited an increased susceptibility to exogenous oxidative stress compared to astrocyte, leading to an increased production of oxidative-stress induced apoptosis in neurons [34]. Other *in vivo* studies of pregnant ogg1 knockout mice treated with a methamphetamine, which would induce neurotoxicity for increased levels of the oxidative DNA lesion 8-oxoG, showed that they exhibited enhanced levels of 8-oxoG in fetal brain and the null offspring exhibited long-term postnatal neurodevelopmental deficits such as dysfunctional motor coordination [35, 36].

**Fig. 7.**
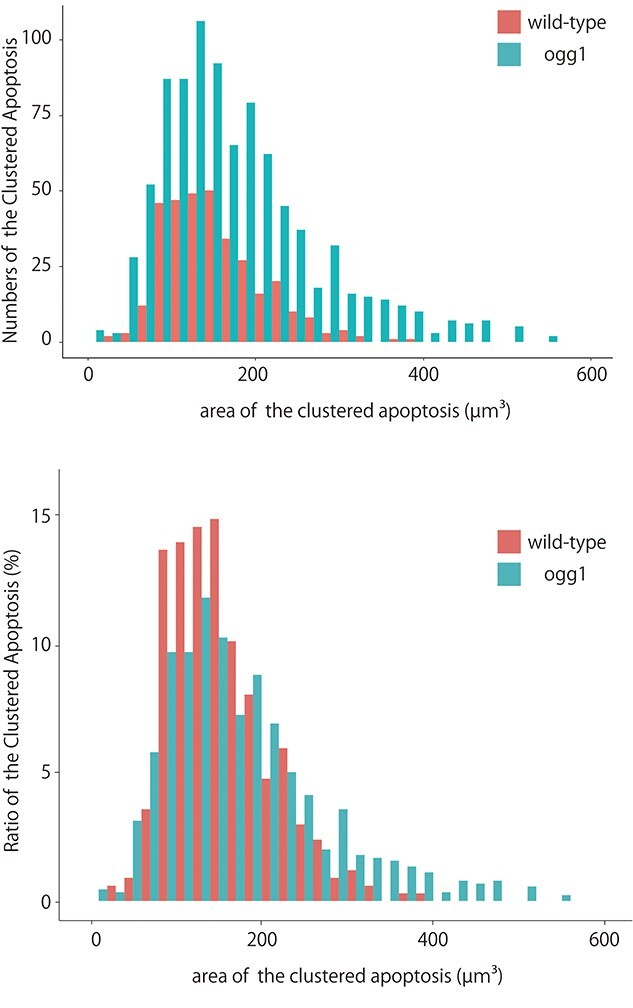
A comparison of the induction frequency and the size of clustered apoptosis between wild-type and ogg1-deficient embryonic brains at 24 h after irradiation. The histograms indicate that the numbers of clustered apoptosis in all sizes, which are classified from 0 to 560 μm^2^ in increments of 20 μm^2^, for ogg1-deficient embryos were larger than those for wild-type embryos (A). The ratios of the area in each size class to the total number of clustered apoptosis (B).

Collectively, these cellular and *in vivo* results including our study focusing on ogg1 provide that 8-oxoG constitutes molecular lesions relating to embryopathy and deficiencies in ogg1 may constitute important risk factors for developmental abnormalities. There is a possibility that irradiated ogg1 deficient embryos exhibit subtle abnormalities in their behaviors for schooling and reproduction when they mature, which should be examined in detail in future studies. Further understanding of the developmental effects of 8-oxoG may provide insights into risk assessment for adverse developmental outcomes and the reduction of adverse postnatal functional deficits and diseases.

## Supplementary Material

suppl_Fig_1_method_3D_rrac005

Suppl_Table_1_rrac005

## References

[1] Ames BN, Shigenaga MK, Hagen TM. Oxidants, antioxidants, and the degenerative diseases of aging. Proc Natl Acad Sci U S A 1993;90:7915–22. 8367443 10.1073/pnas.90.17.7915 PMC47258

[2] Kryston TB, Georgiev AB, Pissis P et al. Role of oxidative stress and DNA damage in human carcinogenesis. Mutat Res 2011;711:193–201. 21216256 10.1016/j.mrfmmm.2010.12.016

[3] David SS, O'Shea VL, Kundu S. Base-excision repair of oxidative DNA damage. Nature 2007;447:941–50. 17581577 10.1038/nature05978 PMC2896554

[4] Hazra TK, Das A, Das S et al. Oxidative DNA damage repair in mammalian cells: a new perspective. DNA Repair (Amst) 2007;6:470–80. 17116430 10.1016/j.dnarep.2006.10.011 PMC2702509

[5] Rosenquist TA, Zharkov DO, Grollman AP. Cloning and characterization of a mammalian 8-oxoguanine DNA glycosylase. Proc Natl Acad Sci U S A 1997;94:7429–34. 9207108 10.1073/pnas.94.14.7429 PMC23838

[6] Klungland A, Bjelland S. Oxidative damage to purines in DNA: role of mammalian Ogg1. DNA Repair (Amst) 2007;6:481–8. 17127104 10.1016/j.dnarep.2006.10.012

[7] Schonfeld P, Reiser G. Why does brain metabolism not favor burning of fatty acids to provide energy? Reflections on disadvantages of the use of free fatty acids as fuel for brain. J Cereb Blood Flow Metab 2013;33:1493–9. 23921897 10.1038/jcbfm.2013.128 PMC3790936

[8] Sykora P, Wilson DM 3rd, Bohr VA. Base excision repair in the mammalian brain: implication for age related neurodegeneration. Mech Ageing Dev 2013;134:440–8. 23643943 10.1016/j.mad.2013.04.005 PMC3834072

[9] Lillenes MS, Rabano A, Stoen M et al. Altered DNA base excision repair profile in brain tissue and blood in Alzheimer's disease. Mol Brain 2016;9:61. 27234294 10.1186/s13041-016-0237-z PMC4884418

[10] Pao PC, Patnaik D, Watson LA et al. HDAC1 modulates OGG1-initiated oxidative DNA damage repair in the aging brain and Alzheimer's disease. Nat Commun 2020;11:2484. 32424276 10.1038/s41467-020-16361-y PMC7235043

[11] Fukae J, Takanashi M, Kubo S et al. Expression of 8-oxoguanine DNA glycosylase (OGG1) in Parkinson's disease and related neurodegenerative disorders. Acta Neuropathol 2005;109:256–62. 15841414 10.1007/s00401-004-0937-9

[12] Cardozo-Pelaez F, Cox DP, Bolin C. Lack of the DNA repair enzyme OGG1 sensitizes dopamine neurons to manganese toxicity during development. Gene Expr 2005;12:315–23. 16358418 10.3727/000000005783992007 PMC6009123

[13] Wells PG, McCallum GP, Chen CS et al. Oxidative stress in developmental origins of disease: teratogenesis, neurodevelopmental deficits, and cancer. Toxicol Sci 2009;108:4–18. 19126598 10.1093/toxsci/kfn263

[14] Branum AM, Collman GW, Correa A et al. The National Children's Study of Environmental Effects on Child Health and Development. Environ Health Perspect 2003;111:642–6. 12676629 10.1289/ehp.111-1241458 PMC1241458

[15] Oberley L, Lindgren A, Baker S et al. Superoxide ion as the cause of the oxygen effect. Radiat Res 1976;68:320–8. 790446

[16] Biaglow JE, Mitchell JB, Held K. The importance of peroxide and superoxide in the X-ray response. Int J Radiat Oncol Biol Phys 1992;22:665–9. 1312073 10.1016/0360-3016(92)90499-8

[17] Wittbrodt J, Shima A, Schartl M. Medaka--a model organism from the far east. Nat Rev Genet 2002;3:53–64. 11823791 10.1038/nrg704

[18] Shima A, Mitani H. Medaka as a research organism: past, present and future. Mech Dev 2004;121:599–604. 15210169 10.1016/j.mod.2004.03.011

[19] Iwamatsu T . Stages of normal development in the medaka Oryzias latipes. Mech Dev 2004;121:605–18. 15210170 10.1016/j.mod.2004.03.012

[20] Yasuda T, Aoki K, Matsumoto A et al. Radiation-induced brain cell death can be observed in living Medaka embryos. J Radiat Res 2006;47:295–303. 16988493 10.1269/jrr.0617

[21] Yasuda T, Oda S, Ishikawa Y et al. Live imaging of radiation-induced apoptosis by yolk injection of acridine orange in the developing optic tectum of Medaka. J Radiat Res 2009;50:487–94. 19680012 10.1269/jrr.09043

[22] Yasuda T, Ishikawa Y, Shioya N et al. Radical change of apoptotic strategy following irradiation during later period of embryogenesis in medaka (Oryzias latipes). PLoS One 2018;13. 10.1371/journal.pone.0201790 PMC6075778 30075024

[23] Ishikawa T, Kamei Y, Otozai S et al. High-resolution melting curve analysis for rapid detection of mutations in a Medaka TILLING library. BMC Mol Biol 2010;11:70. 20840787 10.1186/1471-2199-11-70 PMC2949603

[24] Yasuda T, Yoshimoto M, Maeda K et al. Rapid and simple method for quantitative evaluation of neurocytotoxic effects of radiation on developing Medaka brain. J Radiat Res 2008;49:533–40. 18654045 10.1269/jrr.08030

[25] Yasuda T, Oda S, Hibi Y et al. Embryonic Medaka model of microglia in the developing CNS allowing in vivo analysis of their spatiotemporal recruitment in response to irradiation. PLoS One 2015;10:e0127325. 26061282 10.1371/journal.pone.0127325 PMC4465025

[26] Taniguchi Y, Takeda S, Furutani-Seiki M et al. Generation of medaka gene knockout models by target-selected mutagenesis. Genome Biol 2006;7:1–14. 10.1186/gb-2006-7-12-r116 PMC1794429 17156454

[27] Hyodo-Taguchi Y, Egami N. Establishment of inbred strains of the medaka Oryzias latipes and the usefulness of the strains for biomedical-research. Zool Sci 1985;2:305–16.

[28] Abrams JM, White K, Fessler LI et al. Programmed cell death during drosophila embryogenesis. Development 1993;117:29–43. 8223253 10.1242/dev.117.1.29

[29] Furutani-Seiki M, Jiang YJ, Brand M et al. Neural degeneration mutants in the zebrafish, Danio rerio. Development 1996;123:229–39. 9007243 10.1242/dev.123.1.229

[30] Fiala JC . Reconstruct: a free editor for serial section microscopy. J Microsc 2005;218:52–61. 15817063 10.1111/j.1365-2818.2005.01466.x

[31] Yasuda T, Kimori Y, Nagata K et al. Irradiation-injured brain tissues can self-renew in the absence of the pivotal tumor suppressor p53 in the medaka (Oryzias latipes) embryo. J Radiat Res 2016;57:9–15. 26410759 10.1093/jrr/rrv054 PMC4708913

[32] Gu A, Ji G, Yan L et al. The 8-oxoguanine DNA glycosylase 1 (ogg1) decreases the vulnerability of the developing brain to DNA damage. DNA Repair (Amst) 2013;12:1094–104. 24075420 10.1016/j.dnarep.2013.08.018

[33] Zhou T, Zhang J, Qin B et al. Long non-coding RNA NONHSAT143692. 2 is involved in oxidative DNA damage repair in the lens by regulating the miR-4728-5p/OGG1 axis. Int J Mol Med 2020;46:1838–48. 33000245 10.3892/ijmm.2020.4707 PMC7521474

[34] Harrison JF, Hollensworth SB, Spitz DR et al. Oxidative stress-induced apoptosis in neurons correlates with mitochondrial DNA base excision repair pathway imbalance. Nucleic Acids Res 2005;33:4660–71. 16107556 10.1093/nar/gki759 PMC1187820

[35] Wong A, Jeng W, Wells P. Methamphetamine-initiated neurodevelopmental deficits are enhanced in oxoguanine glycosylase 1 (ogg1) knock-out mice. Toxicol Sci 2004;78:380.

[36] Jeng W, Wong AW, Ting AKR et al. Methamphetamine-enhanced embryonic oxidative DNA damage and neurodevelopmental deficits. Free Radic Biol Med 2005;39:317–26. 15993330 10.1016/j.freeradbiomed.2005.03.015

