# Supplementary figures and images for: 3D reconstructed brain images reveal the possibility of the ogg1 gene to suppress the irradiation-induced apoptosis in embryonic brain in medaka (Oryzias latipes)
Source: J Radiat Res. 2022 Mar 12;63(3):319–30. doi: 10.1093/jrr/rrac005 (PMC9124622; doi:10.1093/jrr/rrac005)

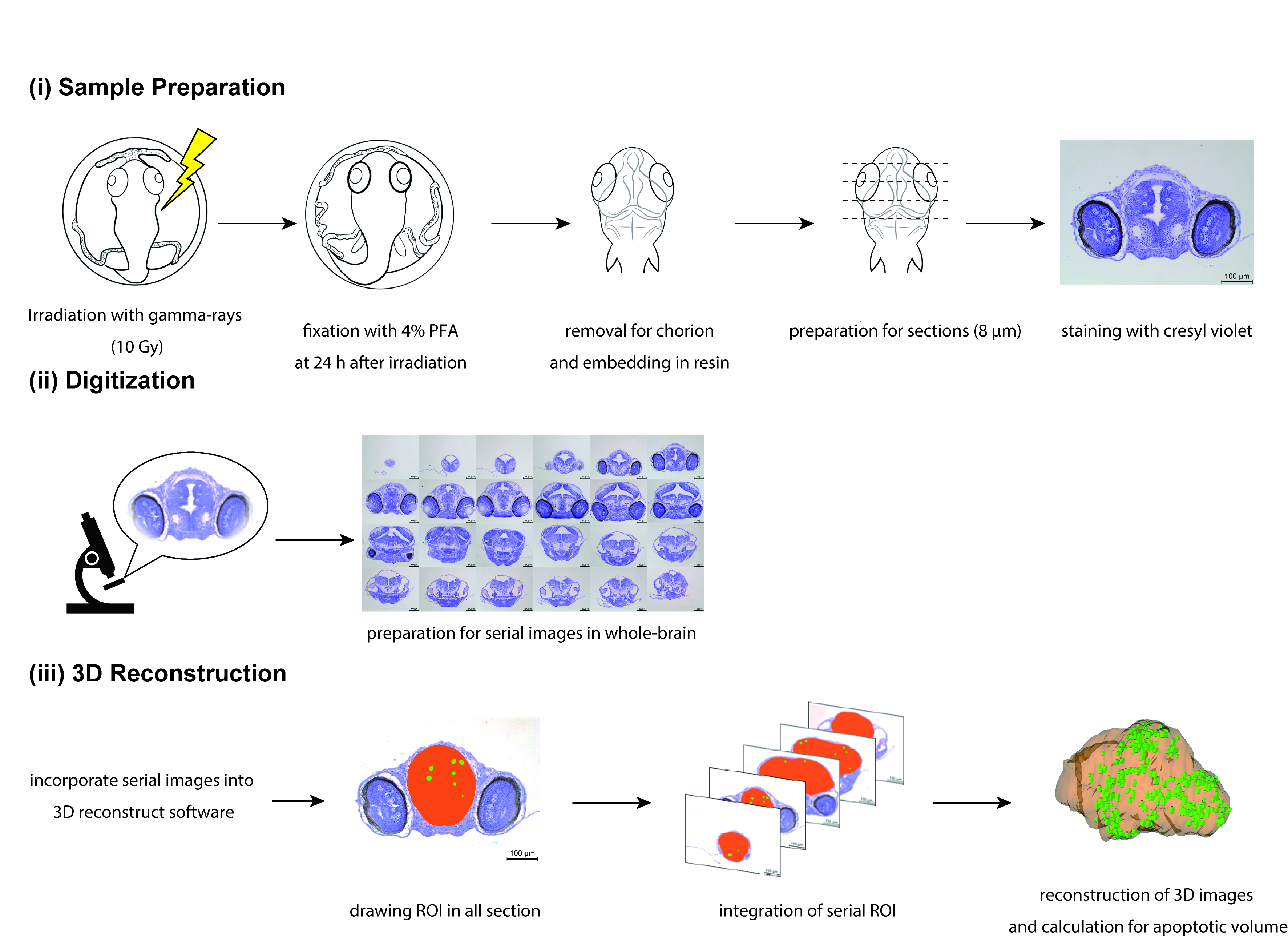

Supplement: suppl_Fig_1_method_3D_rrac005 [file suppl_fig_1_method_3d_rrac005.zip › suppl_Fig_1_method_3D_rrac005.tif]
