# Supplementary material for: 3D reconstructed brain images reveal the possibility of the ogg1 gene to suppress the irradiation-induced apoptosis in embryonic brain in medaka (Oryzias latipes)
Source: J Radiat Res. 2022 Mar 12;63(3):319–30. doi: 10.1093/jrr/rrac005 (PMC9124622; doi:10.1093/jrr/rrac005)
Supplement: Suppl_Table_1_rrac005 [file suppl_table_1_rrac005.docx]

|  |  | Volume of  Clustered Apoptosis | Brain Volume |
| --- | --- | --- | --- |
| wild-type_IR/wild-type_control | fold change | 91.22 | 0.88 |
|  | p-value | 0.06 | 0.10 |
| ogg1_IR/ogg1_control | fold change | 77.30 | 0.75 |
|  | p-value | 0.01 | 0.06 |
| ogg1_IR/wild-type_IR | fold change | 3.13 | 0.58 |
|  | p-value | 0.05 | 0.01 |
| ogg1_control/wild-type_control | fold change | 3.70 | 0.68 |
|  | p-value | 0.11 | 0.004 |

Suppl. Table 1
